# Unlocking the Potential: Amino Acids’ Role in Predicting and Exploring Therapeutic Avenues for Type 2 Diabetes Mellitus

**DOI:** 10.3390/metabo13091017

**Published:** 2023-09-15

**Authors:** Yilan Ding, Shuangyuan Wang, Jieli Lu

**Affiliations:** 1 Department of Endocrine and Metabolic Diseases, Shanghai Institute of Endocrine and Metabolic Diseases, Ruijin Hospital, Shanghai Jiao Tong University School of Medicine, Shanghai 200025, China; dingyilan@sjtu.edu.cn (Y.D.); wsy12060@rjh.com.cn (S.W.); 2 Shanghai National Clinical Research Center for Endocrine and Metabolic Diseases, Key Laboratory for Endocrine and Metabolic Diseases of the National Health Commission of the PR China, Shanghai National Center for Translational Medicine, Ruijin Hospital, Shanghai Jiao Tong University School of Medicine, Shanghai 200025, China

**Keywords:** amino acids, type 2 diabetes mellitus, prediction, mechanism, intervention

## Abstract

Diabetes mellitus, particularly type 2 diabetes mellitus (T2DM), imposes a significant global burden with adverse clinical outcomes and escalating healthcare expenditures. Early identification of biomarkers can facilitate better screening, earlier diagnosis, and the prevention of diabetes. However, current clinical predictors often fail to detect abnormalities during the prediabetic state. Emerging studies have identified specific amino acids as potential biomarkers for predicting the onset and progression of diabetes. Understanding the underlying pathophysiological mechanisms can offer valuable insights into disease prevention and therapeutic interventions. This review provides a comprehensive summary of evidence supporting the use of amino acids and metabolites as clinical biomarkers for insulin resistance and diabetes. We discuss promising combinations of amino acids, including branched-chain amino acids, aromatic amino acids, glycine, asparagine and aspartate, in the prediction of T2DM. Furthermore, we delve into the mechanisms involving various signaling pathways and the metabolism underlying the role of amino acids in disease development. Finally, we highlight the potential of targeting predictive amino acids for preventive and therapeutic interventions, aiming to inspire further clinical investigations and mitigate the progression of T2DM, particularly in the prediabetic stage.

## 1. Introduction

Diabetes mellitus is a prevalent chronic metabolic disorder characterized by disrupted glucose homeostasis resulting in hyperglycemia [1]. It can be attributed to the progressive impairment of pancreatic beta cell function or the development of insulin resistance, leading to inadequate insulin action [2,3]. The global prevalence of diabetes is estimated to affect over 643 million individuals by 2030, with approximately 6.7 million deaths attributed to diabetes or its complications [4]. Type 2 diabetes mellitus (T2DM) is the predominant subgroup of diabetes, accounting for at least 90% of cases worldwide [5]. T2DM significantly impacts quality of life and life expectancy, and is a major contributor to disability or mortality [6]. Chronic exposure to hyperglycemia gives rise to numerous detrimental clinical consequences, including microvascular and macrovascular complications such as nephropathy, retinopathy and neuropathy [7]. Hence, a comprehensive understanding of the metabolic disturbances in T2DM is crucial for effective management.

Amino acids serve as fundamental building blocks for proteins and peptides, possessing both carboxylic and amino functional groups. Beyond their role in protein synthesis, these molecules play critical roles in various cellular processes, including cell signaling, oxidative stress, nutrition metabolism or maintenance [8]. Recent cross-sectional or prospective studies have shed light on the important involvement of amino acids in the development of T2DM [9] and insulin resistance [10]. Amino acids are closely associated with glucose dysregulation, particularly in their role as a primary source for gluconeogenesis, a process wherein glucose is synthesized from non-carbohydrate sources. The over-activation of gluconeogenesis is a key factor in prediabetes, which precedes the onset of T2DM [11]. Disturbances of amino acid metabolism may break the balance of muscle breakdown, protein synthesis and gluconeogenesis in the liver and kidneys. Under specific circumstances, amino acids may enhance glucose-stimulated insulin secretion or modulate insulin sensitivity early in the pathogenesis of T2DM [12]. The response effects are complex and depend on the different types of amino acids [13]. In addition to from the impairment of insulin sensitivity, certain altered amino acids could also block insulin signaling and affect lipid metabolism and mitochondrial oxidation. These changes have initiating and causal roles before insulin resistance is established. As T2DM advances and insulin resistance worsens, the combination of impaired beta cell function and resistance may lead to accelerated muscle proteolysis and disturbances in metabolic signals, further contributing to amino acid dysmetabolism [14]. These findings reveal the interconnectedness of amino acid metabolism and prediabetes, underscoring the great potential of amino acids in the early detection of metabolic abnormalities and prediction of future T2DM.

This review provides a comprehensive description of amino acids as strong correlative factors and mechanistic implications for insulin resistance, prediabetes, and future incident T2DM. Furthermore, we explore the promising inventions targeting amino acids, aiming to inspire advances in clinical research and therapeutic strategies for T2DM.

## 2. The Interrelation between Amino Acids and T2DM

The roles and underlying mechanisms of various amino acids in relation to T2DM represent a prominent area of investigation within the field of glucose metabolism regulation. The dysregulation of amino acid metabolism is crucial in prediabetes and future diabetes onset risk. The following sections elaborate the interrelation between several amino acids and T2DM, including branched-chain amino acids, aromatic amino acids, tryptophan, glycine, asparagine and aspartate.

### 2.1. Branched-Chain Amino Acids

#### 2.1.1. Branched-Chain Amino Acid Metabolism in Health and T2DM

Branched-chain amino acids (BCAAs), including leucine, isoleucine and valine, are often studied as a collective unit. They share structural characteristics with a branched-side chain and undergo the common initiation steps of catabolism. BCAAs cannot be synthesized in higher organisms, making them nutritionally essential amino acids derived from protein-containing foods [15,16]. Through a specialized signaling network, BCAAs significantly modulate and regulate various metabolic and physiological processes, such as glucose, lipid or energy homeostasis [17]. 

Consistent correlations between elevated plasma BCAAs and T2DM have been observed in both human [18,19] and rodent models [20,21]. In the early 1970s, elevated plasma BCAAs were first described in diabetic patients with impaired insulin signaling [22]. In 2011, a prospective cohort study conducted within the Framingham Offspring Study revealed highly significant associations between baseline plasma BCAAs and future diabetes risk among 2422 normoglycemic individuals followed for 12 years. These results were further validated in many independent, prospective cohorts [23,24]. We conducted a nested case-control study on 3414 normoglycemic Chinese populations from a nation-wide prospective cohort of the China Cardiometabolic Disease and Cancer Cohort (4C) to explore the link between amino acids and incident diabetes. The results showed that higher levels of BCAAs were significantly associated with an increased risk of type 2 diabetes mellitus (T2DM) after accounting for various factors. Additionally, we found that triglycerides (TG) and waist-to-hip ratio (WHR) partially mediated the association between BCAAs and incident T2DM, providing further insights into the underlying mechanisms [23].A case-cohort study, including a random sample of 694 participants from PREDIMED trial, revealed that increases in the BCAA score at 1 year were correlated with a higher T2DM risk in the control group, with hazard ratio (HR) per SD = 1.61 (95% CI 1.02, 2.54) [25,26]. However, when participants were treated with a Mediterranean diet rich in extra-virgin olive oil, they experienced decreased BCAA levels, which attenuated the positive association between BCAAs and T2DM incidence [25]. Recent large prospective and cross-sectional cohort studies have also revealed close associations between elevated blood levels of BCAAs and insulin resistance, homeostasis model assessment (HOMA) insulin sensitivity and HbA1c level [12,27,28,29]. Higher plasma BCAA levels were found to be inversely correlated with insulin sensitivity but positively associated with fasting insulin levels [30]. These consistent results have led to speculation about a potential causative role for BCAAs [31].

Despite an overall consistency, varied effect sizes are observed based on age, gender or ethnicity [32]. Generally, BCAAs exhibit higher levels and a closer relationship with T2DM in male participants due to the catabolic differences in the liver [33,34,35]. Fewer studies have investigated the correlations between BCAA concentrations and adverse metabolic outcomes in children and adolescents. Nevertheless, in view of growth hormone secretion and protein turnover during pubertal growth, statistically significant associations have been identified between BCAA levels and future insulin resistance in a pediatric population [36]. Furthermore, individual BCAAs exhibit varying predictive capabilities for future T2DM among different populations [37]. In comparison, valine often stands out in the Chinese population compared to participants of South Asian descent [38]. Presumably, this can be attributed to specific genetic loci, along with earlier beta-cell dysfunction in diabetic patients in China [39]. BCAAs also showed significant differences in the associations with glycemic index and insulin resistance among ethnic groups [40]. African Americans generally exhibit greater insulin resistant, low muscle mass, and higher central obesity, which may explain their higher levels of valine and leucine compared with Hispanics [41].

#### 2.1.2. Mechanisms Underlying Branched-Chain Amino Acids in T2DM 

**Figure 1:**
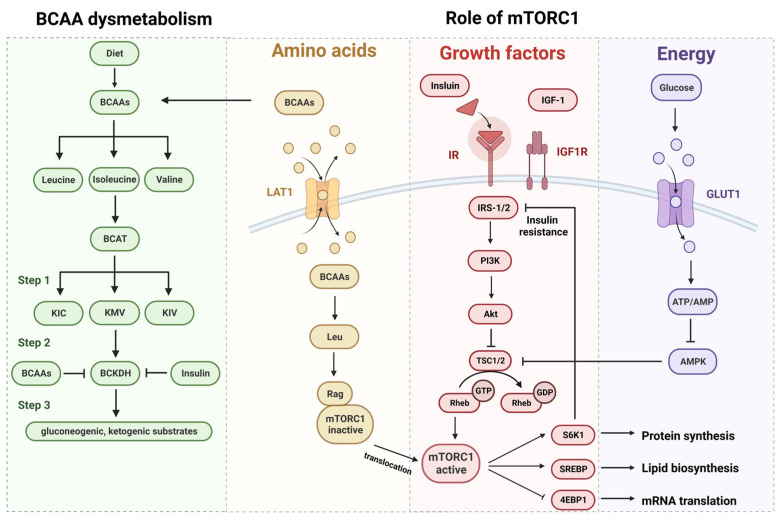
Mechanisms underlying branched-chain amino acids in T2DM. The figure depicts several acknowledged hypothesized mechanisms explaining how BCAAs might contribute to insulin resistance and T2DM. This figure was created with BioRender.com.

Several mechanisms indicate a direct link between BCAAs and, as leucine and isoleucine exhibit insulinotropic effects, while valine and isoleucine are gluconeogenic. Leucine can affect insulin receptor function via the activation of the mammalian target of rapamycin complex 1 (mTORC1), and insulin mediates the branched-chain α-ketoacid dehydrogenase complex (BCKDH). Despite emerging evidence supporting the predictive capability of increased BCAAs levels in T2DM, researchers still contemplate whether BCAAs are true causative factors in insulin resistance and T2DM or merely passive biomarkers of impaired insulin action [27]. Several acknowledged hypothesized mechanisms explaining how BCAAs might contribute to insulin resistance and T2DM are depicted in Figure 1. 

##### Role of mTORC1

One speculated mechanism focuses on the leucine-mediated activation of mTORC1, which leads to the uncoupling of insulin signaling at an early stage. The mammalian target of rapamycin (mTOR) is a serine/threonine kinase that belong to the phosphoinositide 3-kinase (PI3K)-related kinase family and interacts with a series of proteins to form two distinct complexes named mTORC1 and mTORC2 [42]. mTORC1 promotes cell growth, such as protein synthesis, and drives cell cycle progression in response to various stimuli, including growth factors, stress, energy status, oxygen and amino acids. Amino acids, particularly leucine, regulate Rag guanine nucleotide binding, managing the interaction between Rag GTPases and mTORC1 [43]. Various growth factors and signaling molecules regulate the nucleotide state of the small GTPase Rheb (GDP- versus GTP-bound), activating mTORC1-dependent phosphorylation [44]. mTORC1 directly phosphorylates the translational regulators’ eukaryotic translation initiation factor 4E (eIF4E)-binding protein 1 (4E-BP1) and S6 kinase 1 (S6K1), accelerating protein synthesis [45]. Several pathways converge on the tuberous sclerosis complex (TSC) protein, the major upstream negative mediator, to regulate mTORC1 in response to growth factors or cellular stress signaling, notably the prominent upstream growth factor/PI3K/AKT signaling [46]. Apart from the input of growth factor signaling, the 5′ AMP-activated protein kinase (AMPK) signaling is activated and suppresses mTORC1 through activating the phosphorylation of TSC and enhancing its activity during states of energy deficiency [47].

mTOR is a crucial regulator of cellular metabolism and catabolism, while the deregulation of mTOR signaling can induce many human diseases, including diabetes, degenerative disorders and cancer [48]. Persistent nutrient signaling results in insulin resistance by BCAA activation of the mTORC1 signaling pathway. The mTORC1-S6K-mediated negative feedback loops have a deleterious effect on the regulation of insulin signaling, maintaining beta cell function and survival [49]. Continuous stimulation of the serine kinases S6K1 and mTORC1 induces insulin resistance through the recruitment and phosphorylation of insulin receptor substrate (IRS)-1 and IRS-2 at multiple tyrosine residues [50]. These sites function as docking motifs for PI3K and the subsequent phosphorylation of Akt, which disrupts its interaction with insulin signaling. This negative feedback loop attenuates insulin responses, resulting in a reduction in glucose utilization. Under a chronic diabetic milieu, the overwhelming demand for insulin invites impaired insulin action and the potentiation of beta cell dysfunction, resulting in T2DM eventually becoming evident [27]. 

##### BCAA Dysmetabolism

The second hypothetical mechanism analyses how BCAA dysmetabolism generates insulin resistance and T2DM. This hypothesis derives from studies of maple syrup urine disease (MSUD) and organic acidurias, which are inborn errors in metabolism caused by defects in BCKDH, leading to the elevation of BCAAs in plasma and α-ketoacids in urine [51]. 

BCAAs are imported into cells by L-type amino acid transporters (LATs). BCAA catabolism involves three main steps. Firstly, intracellular BCAAs are converted to branched-chain α-keto acids (BCKAs), including α-ketoisocaproate (KIC), α-keto-β-methylvalerate (KMV), and α-ketoisovalerate (KIV). BCAA transaminase (BCAT) catalyzes the transamination of corresponding BCKAs. Then, BCKAs undergo decarboxylation and dehydrogenation to yield respective ketoacids via BCKDH. These first two steps are shared by all three BCAAs, while the latter process is rate-controlling. Numerous metabolic factors altered in insulin resistance and T2DM impair BCAA catabolism by coordinating the downregulation of multiple enzymes, including BCAT and BCKDH. Insulin can directly inhibit BCKDH under insulin-resistant states, giving rise to elevated BCAA and BCKAs levels that are widely believed to be the toxic factors in the disease. Furthermore, elevated plasma BCAA levels can also arise from protein breakdown and degradation in the body [52,53]. The administration of BCKDH kinase (BDK) inhibitors might impair glucose tolerance and reduce plasma BCAA concentrations in rats, independent of the action of insulin [54]. When fed a BCAA-supplemented diet, spontaneous type 2 diabetes Otsuka Long-Evans Tokushima Fatty (OLETF) rats showed improved glucose tolerance upon repeated administration [55]. Animal models discussed the effects of administration of BCAAs, but data in humans are lacking. However, based on observational study, elevated BCAA levels can be perceived long before the occurrence of insulin resistance and seemingly contribute to insulin resistance and T2DM. It is reasonable that BCAAs give rise to an altered insulin-regulated metabolism in the early stages, while insulin, in turn, incurs the accumulation of BCAAs in the later stages of insulin resistance [56]. An impaired BCAAs metabolism induces higher levels of BCAAs and the accumulation of toxic metabolites, leading to mitochondrial bioenergetic dysfunction and subsequent apoptosis of beta cells [27]. It has been confirmed that individuals or animal models with impaired or incomplete BCAA metabolism could be more susceptible to insulin resistance or T2DM [57]. 

Moreover, BCAA dysmetabolism is involved in the regulation of macrophage activity in the onset of chronic low-grade inflammation under diabetic state. BCAA oxidative defects may promote inflammatory response and organ damage in T2DM conditions by inducing macrophage activation [58]. BCKAs can significantly increase the production of detrimental mediators such as ROS, cytokines, and chemokines in primary macrophages. It is demonstrated that BCKA stimulation could alter the expression of a key glucose transporter (Glut1) and enhance the utilization of glucose for ROS overproduction in macrophages via glut1-mediated glucose metabolism. Elevated glycolysis induced ROS-driven proinflammatory phenotype in macrophages, accelerating the promotion of insulin resistance [59]. Additionally, BCKAs also enhance cytokine release by incurring mitochondrial oxidative stress in macrophages. These findings reveal the possible mechanisms by which BCAA dysmetabolism plays an integral role in insulin resistance and T2DM. 

### 2.2. Aromatic Amino Acids

#### 2.2.1. Phenylalanine and Tyrosine Metabolism

Aromatic amino acids (AAAs) are precursors of many significant biological compounds and are necessary for the normal functioning of the human organism. Two kinds of AAAs, phenylalanine and tyrosine, have been observed to be connected with a tendency of increased risk of T2DM [41]. Mice fed with diets rich in phenylalanine could develop insulin resistance and T2DM symptoms [59]. Furthermore, changes in phenylalanine and tyrosine levels closely parallel the changes in fasting blood glucose (FPG) and 2 h postprandial blood glucose (2hPG) levels of individuals [60]. These two AAAs were also demonstrated to be elevated in subjects who developed T2DM from normal glucose tolerance. The levels of phenylalanine and tyrosine increased in hyperglycemia and particularly affected non-diabetic participants, who later developed T2DM over a 5-year follow-up period [41]. Using a nested case-control design from the China Cardiometabolic Disease and Cancer Cohort (4C) Study, our team also revealed that per SD increments in two AAAs had strong associations with the onset of T2DM [23]. Phenylalanine and tyrosine were positively correlated with T2DM, with a 23% increased risk of incident diabetes in the fully adjusted model including all the confounding factors, including diet score, liver enzymes, 2hPG, and HOMA-IR. These results align with previous findings, confirming the predictive value of two AAAs for risk of diabetes in normoglycemic Chinese individuals. AAAs have important implications for the pathogenesis of diabetes. It has been reported that phenylalanine modified insulin receptor beta (IRβ) and inhibited insulin signaling and glucose uptake [61]. Using phenylalanine and aspartame to mimic extremes for serum phenylalanine elevation in humans, phenylalanyl-tRNA synthetase (FARS) sensed phenylalanine concentrations and converted them into the phenylalanine signal by modifying proteins. For IRβ, the phenylalanine signal led to impairments in the components of the insulin signaling cascade and hindered glucose uptake by cells. This disruption of insulin signaling transmission by modifying IRβ has adverse effects on insulin sensitivity and accelerates T2DM progression. Tyrosine is involved in gluconeogenesis and glucose transport [62]. Superfluous tyrosine can be rapidly catabolized, weakening the clearance of blood glucose and enhancing gluconeogenesis. Free tyrosine may combine free radicals forming 3-nitrotyrosine, a more cytotoxic mediator that injuries pancreatic islet beta cells. In the case of accelerated rates of oxygen radical and nitric oxide generation in beta cells, insulin could be a potential target [63]. Interaction with insulin affects the receptor binding and hypoglycemic capacities. This research sheds light on the dysmetabolism of two AAAs and the disturbed insulin signaling pathway, leading to a higher risk of T2DM.

#### 2.2.2. Tryptophan Metabolism

##### Tryptophan Metabolism in Health and T2DM 

**Figure 2:**
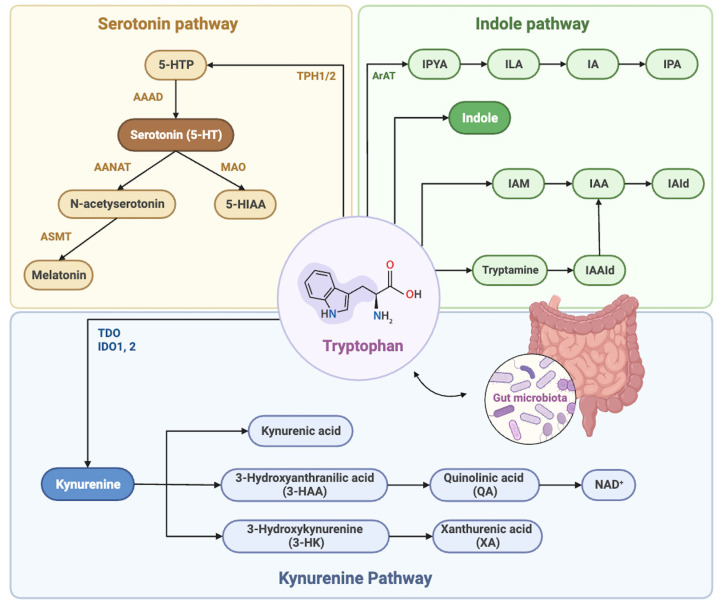
Overview of tryptophan metabolism via the kynurenine, serotonin, and indole pathways. The figure depicts three pathways of tryptophan metabolism including the kynurenine, serotonin, and indole. The gut microbiome can mediate three pathways of tryptophan metabolism and produce correlative metabolites. This figure was created with BioRender.com.

Tryptophan is an indispensable and essential amino acid that can only be obtained through the diet. Systemic and cellular concentrations of tryptophan mainly depend on the balance between biological conversion and degradation pathways. The tryptophan metabolism generally involves three metabolic pathways: the kynurenine (KYN) pathway, the 5-hydroxytryptamine (HT) pathway, and the indole pathway [64]. Most blood tryptophan is bound to albumin and not present in its free form. The majority of free tryptophan in humans is metabolized through the tryptophan–kynurenine pathway, which is involved in extensive physiological functions such as immune activation, growth and feed intake, and alterations in peripheral tissue conditions during aging, obesity and diabetes [65]. The first and rate-limiting step of the tryptophan–kynurenine pathway is catalyzed by the enzyme indoleamine 2,3-dioxygenase (IDO1) and tryptophan-2,3-dioxygenase (TDO). IDO1 exists in most cells, such as macrophages or central nervous cells, while TDO is almost exclusively expressed in the liver and mainly controls tryptophan concentrations in the blood [66]. Chronic stress or inflammatory factors activate enzymes of the upstream steps of tryptophan metabolism and convert tryptophan into KYN and KYN into kynurenic acid (KYNA), 3-hydroxykynurenine (3-HK) and anthranilic acid (AA). The further conversion of 3-HK to 3-hydroxyanthranilic acid (3-HAA) and alanine is catalyzed by kynureninase (KYNU), whereas xanthurenic acid (XA) is another conversion of 3-HK. Subsequently, 3-HAA transforms into the neurotoxic quinolinic acid (QA) and is also crucial in the production of the coenzyme NAD^+^, contributing to energy metabolism and mitochondrial functions. Three pathways of tryptophan metabolism including the kynurenine, serotonin, and indole are depicted in Figure 2.

The gut microbial function in the tryptophan metabolism has emerged as a vital driving force [67]. The gut microbiome can mediate three pathways of tryptophan metabolism and produce correlative metabolites [68]. Tryptophan and its metabolites serve as critical communication regulators between the host and gut microorganisms, maintaining metabolic homeostasis. In germ-free mice, the KYN pathway was inhibited, leading to decreased tryptophan levels. However, after supplementation with intestinal flora, this KYN pathway was normalized [69]. In addition, several metabolites produced by gut microbes play a significant role in adjusting the tryptophan–kynurenine pathway, including the inhibition of IDO transcription [70].

Recent metabolomics screens have confirmed tryptophan metabolites as potential biological mediators in the onset of T2DM [71]. A ten-year longitudinal Shanghai Diabetes Study (SHDS) with 213 participants claimed that serum tryptophan level was significantly higher in individuals who developed future T2DM and was positively and independently related to diabetes onset risk [72]. Higher tryptophan concentration could adversely contribute to a higher degree of insulin resistance and secretion, triglyceride level and blood pressure.

Generally, diabetics often show elevated tryptophan metabolism with decreased tryptophan and increased concentrations of downstream metabolites along the tryptophan–kynurenine pathway [73]. Cross-sectional studies have confirmed increased plasma levels of KYN and KYNA in subjects with insulin resistance prior to the evident manifestation of hyperglycemia and lower levels of tryptophan in nondiabetics [74]. In a metabolomics study including 5181 participants from the cross-sectional Metabolic Syndrome in Men study, the levels of KYNA and other downstream metabolites weakened insulin secretion and insulin sensitivity but enhanced susceptibility to T2DM [75]. In our prospective cohort study, in the fully adjusted model with all confounding factors, serum N-acetyltryptophan, but not tryptophan or kynurenine, was associated with increased risk of diabetes [23]. On this basis, another study involving 2519 individuals with coronary artery disease (CAD) but without T2DM focused on tryptophan and its downstream metabolite kynurenine for a median of 7.6 years [76]. The plasma and urine kynurenine-to-tryptophan ratio (KTR) provided a more suitable measure of tryptophan catabolism than the absolute level of kynurenine or tryptophan. It was observed that KTR in urine, but not in plasma, had a strong positive relationship with incident T2DM during 7 years of follow-up in this large cohort. In two cohorts comprising 856 individuals with T2DM, the serum KTR was associated with and improved the prediction of all-cause mortality among patients [77].

##### Mechanisms Underlying Tryptophan in T2DM

Various observational clinical studies analyzing clinical parameters in the tryptophan–kynurenine pathway reveal the existence of disturbed tryptophan metabolism in prediabetes or diabetes. However, these studies have not definitively determined whether the changes are causative or secondary to the disorder [78]. The diabetogenic tryptophan–kynurenine pathway is affected by many factors, including genetic factors, metabolic status, the degree of pancreas destruction and insulin resistance. These findings underline the potential value of tryptophan and downstream metabolites in identifying high-risk individuals before the occurrence of T2DM, even before remarkable alterations in metabolic markers are observed. However, the mechanisms behind the effects of the tryptophan pathways on T2DM are diverse. In cases of inflammation or stress, the KYN and KYN-NAD metabolic pathways particularly rely on pyridoxal-5-phosphate (P5P), an active form of vitamin B6, as a cofactor. The relative absence of P5P shifts the KYN-NAD metabolism from the common production of NAD^+^ to the excessive formation of XA [79]. The accumulation of XA or other KYN metabolites have diabetogenic effects and can impair the biological function of insulin, promoting the progression of T2DM from prediabetes [80]. Additionally, systemic low-grade inflammation trigged by the dysregulation of tryptophan metabolism can lead to insulin resistance [79]. The severity of insulin resistance varies with central and peripheral concentrations of tryptophan and downstream metabolites. These metabolites can form less active chelate complexes with insulin and interfere with the glucose regulatory network at the prediabetic stage [74]. Serum tryptophan levels may initially increase during prediabetes and then gradually diminish with the advent of a full diabetic state. Nonetheless, monitoring of the tryptophan–kynurenine pathway, especially the KTR, is beneficial in recognizing individuals at risk for T2DM. 

### 2.3. Glycine Metabolism

#### 2.3.1. Glycine Metabolism in Health and T2DM

Glycine is a kind of nonessential amino acid in humans or other mammals. It is associated with various metabolic pathways and involved in numerous human physiological processes. Generally, the amount of glycine synthesized in vivo is insufficient to satisfy metabolic demands. A brief period of glycine shortage may not be a great hazard to health status while chronic depletion can affect growth, immune responses or health metabolism. Glycine usually functions as a precursor for various crucial metabolites of low molecular weight like glutathione synthesis or as a regulator of protein configuration and activity [81]. 

Decreased plasma glycine concentration is now regarded as a promising predictive factor for reduced glucose tolerance and T2DM. Prospective studies demonstrate that higher serum glycine level indicates a reduced risk of incident T2DM and hypoglycemia at baseline can suggest an inclination for T2DM. Patients with obesity or diabetes observed a relative lower plasma glycine concentration compared with control subjects [82]. Particularly, this metabolic change occurs before obvious clinical manifestations of the disorder. Moreover, the level of plasma glycine correlates positively with insulin sensitivity but negatively with insulin resistance in view of the homeostasis model assessment for the beta cell function index [83]. A randomized trial also reveals that diabetic patients treated with insulin sensitizer therapy including pioglitazone and metformin have higher plasma glycine in comparison to placebo [84]. However, in the prospective cohort, a 4C study did not discover remarkable changes in the ORs of glycine before onset of glucose dysregulation [23]. This calls for further studies to explore the potential usefulness of glycine as a clinical diagnostic tool for T2DM.

#### 2.3.2. Mechanisms Underlying Glycine in T2DM

The pathophysiological mechanisms behind glycine insufficiency and the homeostasis of glycine and T2DM still need to be elaborated. A variety of hypotheses are widely accepted. First and foremost, glycine can directly adjust insulin secretion and has been identified as the strongest amino acid related to increased insulin sensitivity [85]. A positive feedback loop exists between human islet beta cells expressing glycine receptors and insulin depending on phosphoinositide 3-kinase [86]. In liver from sucrose-fed rats, glycine also diminishes the insulin-induced phosphorylation of insulin receptor substrate-1 in serine residue and enhances the phosphorylation of insulin receptor β-subunit in tyrosine residue, which elevates insulin sensitivity [87]. In T2DM with chronic low-grade inflammation, where pro-inflammatory markers are uplifted, glycine is certified as a new anti-inflammatory agent for increasing cytokine IL-10 in monocytes and decreasing TNF-alpha in monocytes and Kupffer cells [88]. These results provide clues of the glycine signaling mechanisms of significant metabolic benefits. Moreover, as one precursor amino acid of the antioxidant glutathione (GSH), glycine can directly affect the synthesis rate and availability of GSH. Animals fed with food short in GSH precursor amino acids were proved to suffer from GSH deficiency. In human cells, reduced GSH is one of the most abundant and common substances resisting damage caused by oxidative stress. However, uncontrolled blood glucose levels lead to oxidative stress and reactive oxygen species (ROS) formation, which is far beyond the capacity of GSH-driven antioxidant defense systems [89]. Deficiency of glycine gives rise to the insufficient synthesis of GSH, which fails to combat subsequent diabetic tissue damage. Accordingly, dietary supplementation with enough glycine may reverse the shortage of GSH synthesis and tackle oxidative stress. 

### 2.4. Asparagine and Aspartate

#### 2.4.1. Asparagine Metabolism in Health and T2DM

Asparagine is a kind of glucogenic amino acid whose byproduct, oxaloacetate, can be used in the TCA cycle to synthesize glucose. With metabolism fluctuation, asparagine and aspartate are readily converted to each other by corresponding enzymes and can undergo transamination to form glutamate. Amid the asparagine metabolism, asparagine synthetase (ASNS) catalyzes asparagine synthesis via aspartate, ATP and ammonia as substrates [90]. ASNS is ubiquitous in its organ distribution and highly associated with cellular nutritional imbalances like glucose deficiency and amino acid disturbances [91]. Asparagine and other biologically active molecules have a vital role in cell catabolism and signaling, host anti-oxidative ability, and immunity under physiological and pathological conditions [92]. Controversy has been arisen regarding the relationship between asparagine, aspartate and T2DM. Circulating concentrations of asparagine are correlated with incidence of T2DM. Only rarely has the literature provided connections between plasma asparagine levels and a lower risk for future T2DM. Animal experimental results revealed lower concentrations of asparagine in diabetic rats [93]. Prospective observational studies demonstrated that baseline plasma asparagine was a protective biomarker of diabetes [94]. According to the Framingham Heart Study, asparagine was negatively related to fasting insulin while aspartate was inversely associated with fasting glucose [95]. Additionally, asparagine was proved to be the sole protective and predictable metabolite for T2DM in a subset of 2939 Atherosclerosis Risk in Communities (ARIC) study participants with metabolomics data and without prevalent diabetes [96]. However, in our previous study, serum asparagine was shown to be associated with an increased risk of diabetes in the multivariable-adjusted model in addition to the adjustment of diet score, and in the fully adjusted model [23]. Particularly, a ratio of asparagine to aspartate of >1.5 contributed to the increased risk of T2DM, which could be further elevated by female gender and being >50 years of age [97]. Thus, whether asparagine is protective or diabetogenic is still a subject of debate. 

#### 2.4.2. Mechanisms Underlying Asparagine and Aspartate in T2DM

Findings about the correlation of asparagine and aspartate with the risk of T2DM are inconclusive, and the underlying mechanisms are also conflicting. On the one hand, asparagine can easily turn into aspartate and then undergo a transamination for glutamate, which is also a constituent of the tripeptide glutathione against oxidative stress and chronic diseases [98]. This assumption explains some of the inverse associations with diabetes risk. On the other hand, some results about asparagine are opposite to the protective correlations with T2DM. The discrepancy between different studies may derive from the sampling stage. Before the occurrence of diabetes, asparagine is likely inadequate while persistent adverse stimulation may upregulate ASNS and induce an excess of asparagine and aspartate deficiency in later periods. Thus, the increasing conversion of asparagine to aspartate contributes to lower concentrations of circulating asparagine and onset of hyperglycemia [99]. This might point toward a potential causal association between low plasma asparagine levels and prediabetes. Beyond that, asparagine can also hinder the phosphorylation of AMPK and upregulate mTORC1, causing increased insulin resistance and decreased beta cell reserve [100]. Uncommon asparagine and aspartate homeostasis with a higher risk of T2DM can be greatly amplified by the specific effect of older age and female gender [96]. Females at later stages of adult life are proven to suffer from a lack of estrogen, which is an important regulator of metabolic status [101]. An absence of estrogen can similarly generate insulin resistance, impaired insulin function and beta cell apoptosis [102]. The cooccurrence of abnormal asparagine and aspartate homeostasis and estrogen insufficiency promotes the process of insulin resistance and accelerates the development of T2DM by the AMPK-mTORC1 pathway. Further investigations into the latent molecular mechanisms are warranted for a better understanding of the cause of T2DM and asparagine and aspartate homeostasis.

### 2.5. Serine Metabolism

#### 2.5.1. Serine Metabolism in Health and T2DM

As a type of nutritionally non-essential amino acid (NEAA), serine can be derived from the diet, or synthesized from 3-phosphoglycerate (3-PG) and glycine. Serine metabolism makes profound contributions to many cellular functions, particularly in the turnover of proteins and phospholipids as necessary building blocks in cellular membranes [103]. In addition, L-serine is required to promote the growth and differentiation of neurons. Deficiency in L-serine is highly correlated with the abnormal synthesis of phospholipids like phosphatidylserine (PS) and sphingolipids (SL), which impairs regular functions of the nervous system. The induction of systemic L-serine deficiency is linked to the risk of driving future T2DM and diabetic peripheral neuropathy [104,105]. Evidence that alterations in serine concentration play a role in T2DM is growing [106,107]. Systemic serine deficiency has been demonstrated to coexist with severe obesity, insulin resistance and hyperglycemia in a mouse model of T2DM [104]. Oral serine supplementation can correct the underlying serine deficiency, thus hindering the development of diabetic peripheral neuropathy in diabetic animals. Moreover, prior studies have shown that, compared with non-diabetic controls, plasma serine concentrations are markedly reduced in the T2DM patients [106,108]. Similar research analyzing the levels of amino acids in diabetic patients has also confirmed a decrease in L-serine concentration in blood [107].

#### 2.5.2. Mechanisms Underlying Serine in T2DM

Irregular serine metabolism is believed to contribute to the pathogenesis of T2DM and related complications, although there is no general consensus regarding the potential mechanisms. Serine has been shown to be capable of promoting insulin secretion, increasing insulin sensitivity, and enhancing glucose tolerance [29]. As deoxysphingolipids accumulate under conditions of decreased serine availability, it is hypothesized that cytotoxic deoxysphingolipids may directly compromise pancreatic beta cells. Diabetic patients have significantly higher plasma levels of deoxysphingolipid in comparison with the control group [109]. The increased concentrations of these lipids impairs normal glucose homeostasis and induces beta cell failure in response to chronic hyperglycemia [110]. Nevertheless, further studies are required, since the mechanisms underlying serine in T2DM remain largely unknown.

### 2.6. Amino Acid Combination

To further promote the predictive performance of plasma amino acids, a combination of three amino acids predicting future diabetes has been reported. The top combination of these three amino acids, namely isoleucine, phenylalanine, and tyrosine, has been identified based on robust statistical measures such as the likelihood ratio (LHR) statistic and c-statistic. When compared to the use of a single amino acid, this combination significantly improves the LHR statistic by from +6 to +9 points (*p* < 0.05). However, the incremental improvement is relatively modest when five additional amino acids are included in the combination. Clinical models incorporating this predictive three-amino acid combination have demonstrated a substantially higher risk, from 5- to 7-fold higher, of developing diabetes among individuals in the top quartile, in contrast with those in the lowest quartile (*p* for trend, from 0.007 to 0.0009) [24]. The results have been successfully replicated in an independent, prospective cohort, confirming the reliability and robustness of the predictive value of the three-amino acid combination in identifying individuals at higher risk for future diabetes. Meanwhile, the elevated concentrations of this three-amino acid combination not only serve as predictors of future diabetes but also provide early signals for the subsequent development of cardiovascular disease and its functional consequences during long-term follow-up [111]. 

Moreover, in addition to the three BCAAs mentioned earlier, two aromatic amino acids (AAAs)—phenylalanine and tyrosine—are commonly grouped together to indicate the occurrence and development of T2DM [112]. Experimental and clinical data have suggested five specific amino acids that may serve as effectors in prediabetes status [113]. Previous research demonstrated associations between these five amino acids and incident diabetes, its precursor states and insulin resistance. Individuals with hyperinsulinemia tend to have greater concentrations of AAAs [114]. Prospective cohorts comprising over 3000 participants highlighted that elevated levels of these five elevated amino acids were associated with 60–100% increases in the relative risk of T2DM [24]. When considering factors such as age and gender, this five-amino acid combination was significantly related to HOMA-IR at baseline and for men at 6-year follow-up (odds ratio 2.09 [95% CI 1.38–3.17]; *p* = 0.0005), while, for women, only leucine, valine and phenylalanine predicted HOMA-IRat the 6-year follow-up (*p* < 0.05) [115]. The combined impact of branched-chain amino acids and aromatic amino acids on promoting insulin resistance and future T2DM is most evident in young normoglycemic adults, particularly in male individuals. In light of these findings, instead of screening for individual amino acids, exploring optimal combinations of amino acids that demonstrate a stronger correlation by including or excluding certain amino acids may be a more meaningful approach.

In addition, a series of amino acid combinations exhibit temporal characteristics in predicting diabetes. Previous research has observed that, at various timepoints, specific amino acids undergo changes in their concentrations and metabolic pathways that may be related to the onset and progression of diabetes. These timepoints include but are not limited to various stages such as pre-diabetes, early-stage diabetes, and disease progression. The elevated of BCAAs and AAAs prior to T2DM could potentially predict the onset of incident diabetes years before the clinical T2DM manifestation. When compared with individuals with NGR, participants with impaired fasting glucose (IFG) showed lower levels of leucine while diabetic patients exhibited increased levels of leucine, tyrosine, and asparagine in contrast to IFG patients [116]. The fluctuations in leucine levels might be characteristic of the transition from NGR to prediabetes or differences between individual patients, which still needs further study [116].

Overall, the characteristics of studies investigating associations between amino acids and type 2 diabetes are summarized in Table 1.

## 3. Targeting Predictive Amino Acids for Preventive and Therapeutic Interventions in T2DM

### 3.1. Lifestyle Interventions

In general, lifestyle interventions promoting a healthy diet with a limited intake of high sugar and saturated fat, combined with enhanced physical activity, are known to reduce the risk for T2DM, especially in high-risk individuals [117,118]. However, few studies have investigated how lifestyle interventions influence the relationship between amino acids and insulin resistance or T2DM. Although the altered levels of circulating amino acids can indicate a risk of imminent T2DM, the extent to which this correlation is independent of lifestyle factors needs to be further studied and established.

In experimental models, young and growing mice subjected to a specific decreased consumption of BCAAs exhibited beneficial effects on metabolic health and improved glucose tolerance [119]. This provides valuable insights into the potential of dietary interventions targeting reduced BCAA intake for the treatment of insulin resistance. Furthermore, the Finnish Diabetes Prevention Study (DPS) explored the association between BCAAs and T2DM in trajectory models via a lifestyle intervention setting. The intervention group underwent lifestyle changes that included supervised food intake and increased physical activity, leading to a decrease in BCAA levels and a diminished association with T2DM risk [120]. The intervention goals placed an emphasis on reducing total and saturated fat intake, increasing fiber density in the diet, and promoting moderate physical activity. Similarly, the above-mentioned PREDIMED trial using a Mediterranean diet supplemented with extra-virgin olive oil as the main intervention resulted in reductions in the plasma BCAA and AAA levels, thereby weakening the subsequent risk of T2DM [25,26]. These associations align with a previous study of BCAAs and CVD [121]. The effects of the MedDiet on BCAA concentrations persisted even after adjusting for changes in insulin or HOMA-IR. Further research is needed to gain a comprehensive understanding of these complex biological mechanisms. As the MedDiet does not specifically target the profile of amino acid intake, the most possible explanation for this intervention is that it alleviates the deleterious correlations between BCAAs and AAA on T2DM risk, potentially via downstream pathways or alternative protective mechanisms. Another lifestyle intervention implemented from 2016 to 2018 involving 5% weight reduction and a diet with increased consumption of whole grains, nuts, low-fat dairy, olive and rapeseed oils and decreased intake of snacks, fast foods, red and processed meat [122] led to a noticeable reduction in BCAA concentrations. This suggests that this approach holds promise in preventing or delaying the onset of T2DM. On the other hand, dietary supplementation with certain amino acids like glycine significantly increased insulin responses and led to remarkable decreases in systemic inflammation, highlighting their potential as protective biomarkers in blood glucose control [123]. In a nutshell, current research highlights the therapeutic potential of manipulating lifestyle interventions targeting amino acids for treating insulin resistance and preventing or delaying the future exacerbation of the T2DM pandemic [124]. Assessing the safe limits of amino acid intake may provide a useful metric to determine appropriate dietary amino acid recommendations, especially for individuals susceptible to T2DM or those already in the prediabetic state.

### 3.2. Pharmacologic Treatment Approaches

Compared with lifestyle interventions alone, different pharmacologic treatment approaches are also beneficial to prevent or delay various subgroups of T2DM. The establishment of appropriate pharmacological interventions could ameliorate the amino-acid-driven impairment of cell signaling and maladaptive phenotypes. Diverse amino acid metabolic pathways are likely to become potential targets of pharmacologic therapies. Common glucose-lowering medications including metformin, glipizide or empagliflozin might alter amino acid levels as a downstream consequence [125]. For instance, the effect of metformin dramatically alters the BCAA metabolism. Sustaining treatment with metformin could adjust circulating BCAA levels in a specific manner. Apart from activating AMPK and reducing hepatic gluconeogenesis and blood glucose, metformin also suppressed BCAT2 and BCKDHa mRNA expression, suggesting that metformin could function via downregulating BCAA catabolic enzyme expression or activity [126]. After the administration of glipizide and metformin, levels of branched-chain amino acids and aromatic amino acids experienced acute changes, which reflect an improvement in glycemic metabolism [112]. Low-dose metformin treatment could rectify glucose metabolic imbalance and integrative metabolomics analysis further investigated the elevation of amino acid levels including serine, glycine, glutamate, along with the decrease in aspartate [127,128]. Beyond this, accumulating evidence has demonstrated metabolic effects of the SGLT2 inhibition empagliflozin on increased concentrations of BCAA metabolites such as acylcarnitine [129]. The hypoglycemic agent DPP4 inhibitor, sitagliptin, induced glycemic improvements and led to a remarkable decrease in plasma valine levels [130]. Sitagliptin significantly changed the pattern of amino acids in both mice and T2DM patients [131]. The mechanisms behind these complex associations remain speculative and need further investigation for the development of novel effective pharmacologic T2DM therapies.

## 4. Conclusions and Future Perspectives

Based on flourishing metabolite profiling platforms, a panel of amino acids has been identified to predict the onset of future diabetes. Specific amino acids play crucial parts early in the pathogenesis of incident insulin resistance and future T2DM. As promising predictive metabolites, the better utilization of amino acids contributes to improving early diagnosis and clinical outcomes, allowing for precautionary measures to be taken to avoid complications, and delaying the onset and progression of T2DM. Enhanced insight into amino acid profiling has also increased interest in various inventions, as it holds therapeutic potential for the management of diabetes. To achieve a more personalized and precise control of T2DM, the translation of these insights to clinical application requires several additional steps. First and foremost, it is necessary to clarify whether these relevant amino acids are merely associated with impaired insulin function or can directly give rise to insulin resistance and subsequent T2DM. Furthermore, the mechanisms behind certain amino acids remain controversial and inconclusive, warranting further investigation to build comprehensive theories that elucidate the dominant signaling pathways and biological mechanisms linking amino acids with diabetes risk.

In conclusion, amino acids hold both predictive and therapeutic potential in future T2DM. A thorough understanding of amino acid dysmetabolism in T2DM is essential for the effective screening, diagnosis and prediction of future diabetic complications, allowing clinicians to make informed decisions and benefitting individuals at risk. Once this diagnosis approach passes to the clinical level, it is expected to achieve considerably high detection accuracy and offer more specific therapeutic possibilities for high-risk patients.

## Figures and Tables

**Table 1:** Characteristics of studies investigating associations between amino acids and type 2 diabetes.

Reference	Study Population Location	N, Follow-Up Time	Study Design	Biological Sample	Methods/Tested Amino Acids	Key Findings
Wang TJ et al., 2011 Nat Med [24]	Discovery analyses: Framingham Offspring Study, U.S. Replication analyses: Malmo Diet and Cancer Study, Sweden	Discovery analyses: 189 cases who developed diabetes during a 12-year follow-up period, and 189 propensity-matched controls who did not develop diabetes Replication analyses: 163 cases and 163 controls	Cohort Nested case-control study	Plasma	LC-MS isoleucine, leucine, valine, tyrosine, phenylalanine, tryptophan, arginine, lysine, histidine, aspartate, glutamic acid, asparagine, glutamine, methionine, serine, threonine, alanine, glycine, proline, cis/trans-hydroxyproline, taurine	Increased risk of T2DM (↑): isoleucine, leucine, valine, tyrosine, phenylalanine, sum of isoleucine, tyrosine and phenylalanine. Replication analyses: leucine, valine, tyrosine, and phenylalanine were significantly associated with increased risk of incident diabetes (↑).
Ruiz-Canela M et al., 2018 Diabetologia [25]	PREvención con DIeta MEDiterránea (PREDIMED) trial	251 T2DM, 694 controls (641 non-T2DM and 53 overlapping cases) 3.8 years	Cohort Nested case-control study	Plasma	LC-MS/MS leucine, isoleucine, valine, phenylalanine, tyrosine	Increased risk of T2DM (↑): baseline BCAA (sum of leucine, isoleucine and valine) and AAA (sum of phenylalanine and tyrosine) scores, BCAAs/AAAs, leucine, isoleucine, valine, phenylalanine, tyrosine.
Wang, S. et al., 2022 Cell Rep Med [23]	China Cardiometabolic Disease and Cancer Cohort (4C)	1707 matched case-control pairs with up to 5 years of follow-up	Cohort Nested case-control study	Serum	UPLC-MS/MS Branched-chain AAs, aromatic AAs, asparagine, alanine, glutamic acid, homoserine, 2-aminoadipic acid, histidine, methionine, asparagine, and proline	Increased risk of T2DM (↑): branched-chain AAs, aromatic AAs, asparagine, alanine, glutamic acid, homoserine, 2-aminoadipic acid, histidine, methionine, and proline.
Stancáková A et al., 2012 Diabetes [60]	METabolic Syndrome In Men (METSIM) study	3026 NGT, 4327 IFG, 312 IGT, 1058 IFG + IGT, 646 T2DM 4.7 years	Population-based cohort	Plasma	High-throughput serum nuclear magnetic resonance (NMR) platform alanine, phenylalanine, valine, leucine, isoleucine, tyrosine, histidine, glutamine	Increased risk of T2DM (↑): alanine, leucine, isoleucine, tyrosine, and glutamine predicted incident T2DM, and their effects were largely mediated by insulin resistance (except for glutamine).
Yamada, C. et al., 2015 J Diabetes Investig [40]	Volunteers Japan	94 non-diabetic Japanese men and women	Cross-sectional study	Plasma	LC/MC serine, asparagine, glutamic acid, glutamine, and other 39 amino acids in total	(↑) Positive correlations were observed between HOMA-IR and valine, isoleucine, leucine, tyrosine, phenylalanine and total BCAA concentration.
Floegel A et al., 2013 Diabetes [71]	European Prospective Investigation into Cancer and Nutrition (EPIC)-Potsdam	800 incident T2DM, 2282 controls 7 years	Cohortnested case-control study	Serum	Targeted FIA-MS/MS 163 metabolites (including 14 amino acids)	Decreased risk of T2DM (↓): glycine; increased risk of T2DM (↑): Phenylalanine.
Vangipurapu, J. et al., 2020 Diabetes Care [75]	Metabolic Syndrome in Men (METSIM) study	5169 participants of METSIM having a follow-up of 7.4 years	Population-based cohort	Plasma	UHPLC 86 microbiome-based metabolites	Increased risk of T2DM (↑): xanthurenate, kynurenate as tryptophan-kynurenine downstream metabolites
Menni C et al., 2013 Diabetes [21]	Twins UK	2204 female (115 T2DM, 192 IFG, 1897 control)	Population-based cohort	Plasma	Nontargeted metabolomicsprovider Metabolon, Inc.42 metabolites	Increased risk of T2DM (↑): proline, 3-Methyl-2-oxovalerate, 4-Methyl-2-oxopentanoate, isoleucine, leucine, valine; decreased risk of T2DM (↓): N-acetylglycine, citrulline, dimethylarginine (SDMA + ADMA); increased risk of IFG (↑): 2-hydroxybutyrate (AHB), 3-methyl-2-oxobutyrate, 3-Methyl-2-oxovalerate, 4-methyl-2-oxopentanoate, isoleucine, leucine.
Tianlu Chen et al., 2016 Plos one [72]	Shanghai Diabetes Study (SHDS)	213 NGT 10 years	Cohort population-based cohort	Serum	UPLC-TQ/MS tryptophan	(↑) Serum tryptophan was positively associated with T2DM risk.
Rebholz, C.M. et al., 2018 Diabetologia [96]	Atherosclerosis Risk in Communities (ARIC) study	2939	Cohort population-based cohort	Serum	isoleucine, leucine, valine, asparagine, 3-(4-hydoxyphenyl) lactate	Increased risk of T2DM (↑): isoleucine, leucine, valine; decreased risk of T2DM (↓): asparagine

(↑), positive association (e.g., higher risk); (↓), inverse association (e.g., lower risk) with prediabetes traits or type 2 diabetes.

## Abbreviations

Type 2 diabetes mellitus (T2DM); branched-chain amino acids (BCAAs); homeostasis model assessment (HOMA); mammalian target of rapamycin complex 1 (mTORC1); branched-chain α-ketoacid dehydrogenase complex (BCKDH); phosphoinositide 3-kinase (PI3K); mammalian target of rapamycin (mTOR); eukaryotic translation initiation factor 4E (eIF4E); 4E binding protein 1 (4E-BP1); S6 kinase 1 (S6K1); tuberous sclerosis tumor suppressor complex (TSC); AMP-activated protein kinase (AMPK); insulin receptor substrate (IRS); maple syrup urine disease (MSUD); L-type amino acid transporters (LATs); α-ketoisocaproate (KIC); α-keto-β-methylvalerate (KMV); α-ketoisovalerate (KIV); BCAA transaminase (BCAT); glucose transporter (Glut1); Aromatic amino acids (AAAs); China Cardiometabolic Disease and Cancer Cohort (4C); normal glucose regulation (NGR); insulin receptor beta (IRβ); phenylalanyl-tRNA synthetase (FARS); 5-hydroxytryptamine (HT); kynurenine (KYN); indoleamine 2,3-dioxygenase (IDO1); tryptophan-2,3-dioxygenase (TDO); kynurenic acid (KYNA); 3-hydroxykynurenine (3-HK); anthranilic acid (AA); 3-hydroxyanthranilic acid (3-HAA); kynureninase (KYNU); xanthurenic acid (XA); quinolinic acid (QA); Shanghai Diabetes Study (SHDS); coronary artery disease (CAD); kynurenine-to-tryptophan ratio (KTR); pyridoxal-5-phosphate (P5P); glutathione (GSH); reactive oxygen species (ROS); asparagine synthetase (ASNS); Atherosclerosis Risk in Communities (ARIC); likelihood ratio (LHR); Diabetes Prevention Study (DPS).
